# Relevance of *TMPRSS2*, CD163/CD206, and CD33 in clinical severity stratification of COVID-19

**DOI:** 10.3389/fimmu.2022.1094644

**Published:** 2023-03-08

**Authors:** Silvia Martínez-Diz, Fernando Marín-Benesiu, Ginesa López-Torres, Olivia Santiago, José F. Díaz-Cuéllar, Sara Martín-Esteban, Ana I. Cortés-Valverde, Verónica Arenas-Rodríguez, Sergio Cuenca-López, Patricia Porras-Quesada, Carmen Ruiz-Ruiz, Ana C. Abadía-Molina, Carmen Entrala-Bernal, Luis J. Martínez-González, Maria Jesus Álvarez-Cubero

**Affiliations:** ^1^ Preventive Medicine and Public Health Service, Hospital Universitario Clínico San Cecilio, Granada, Spain; ^2^ GENYO, Center for Genomics and Oncological Research, Granada, Spain; ^3^ Department of Biochemistry, Molecular Biology III and Immunology, Faculty of Medicine, University of Granada, Granada, Spain; ^4^ Casería de Montijo Health Center, Granada Health District, Granada, Spain; ^5^ Loja Health Center, Metropolitan District of Granada, Loja, Spain; ^6^ Immunology Unit, Institute of Regenerative Biomedicine (IBIMER), Center for Biomedical Research Center (CIBM), University of Granada, Granada, Spain; ^7^ LORGEN G.P., PT, Ciencias de la Salud - Business Innovation Centre (BIC), Granada, Spain; ^8^ Biosanitary Research Institute (ibs. GRANADA), University of Granada, Granada, Spain

**Keywords:** biomarkers, cytometry, COVID-19, SNPs, CyTOF

## Abstract

**Background:**

Approximately 13.8% and 6.1% of coronavirus disease 2019 (COVID-19) patients require hospitalization and sometimes intensive care unit (ICU) admission, respectively. There is no biomarker to predict which of these patients will develop an aggressive stage that we could improve their quality of life and healthcare management. Our main goal is to include new markers for the classification of COVID-19 patients.

**Methods:**

Two tubes of peripheral blood were collected from a total of 66 (n = 34 mild and n = 32 severe) samples (mean age 52 years). Cytometry analysis was performed using a 15-parameter panel included in the Maxpar^®^ Human Monocyte/Macrophage Phenotyping Panel Kit. Cytometry by time-of-flight mass spectrometry (CyTOF) panel was performed in combination with genetic analysis using TaqMan^®^ probes for *ACE2* (rs2285666), *MX1* (rs469390), and *TMPRSS2* (rs2070788) variants. GemStone™ and OMIQ software were used for cytometry analysis.

**Results:**

The frequency of CD163^+^/CD206^-^ population of transitional monocytes (T-Mo) was decreased in the mild group compared to that of the severe one, while T-Mo CD163^-^/CD206^-^ were increased in the mild group compared to that of the severe one. In addition, we also found differences in CD11b expression in CD14^dim^ monocytes in the severe group, with decreased levels in the female group (p = 0.0412). When comparing mild and severe disease, we also found that CD45^-^ [p = 0.014; odds ratio (OR) = 0.286, 95% CI 0.104–0.787] and CD14^dim^/CD33^+^ (p = 0.014; OR = 0.286, 95% CI 0.104–0.787) monocytes were the best options as biomarkers to discriminate between these patient groups. CD33 was also indicated as a good biomarker for patient stratification by the analysis of GemStone™ software. Among genetic markers, we found that G carriers of *TMPRSS2* (rs2070788) have an increased risk (p = 0.02; OR = 3.37, 95% CI 1.18–9.60) of severe COVID-19 compared to those with A/A genotype. This strength is further increased when combined with CD45^-^, T-Mo CD163^+^/CD206^-^, and C14^dim^/CD33^+^.

**Conclusions:**

Here, we report the interesting role of *TMPRSS2*, CD45^-^, CD163/CD206, and CD33 in COVID-19 aggressiveness. This strength is reinforced for aggressiveness biomarkers when *TMPRSS2* and CD45^-^, *TMPRSS2* and CD163/CD206, and *TMPRSS2* and CD14^dim^/CD33^+^ are combined.

## Graphical Abstract

Graphical abstract representing the workflow of the present project and summarizing the main techniques applied to the analysis of samples.
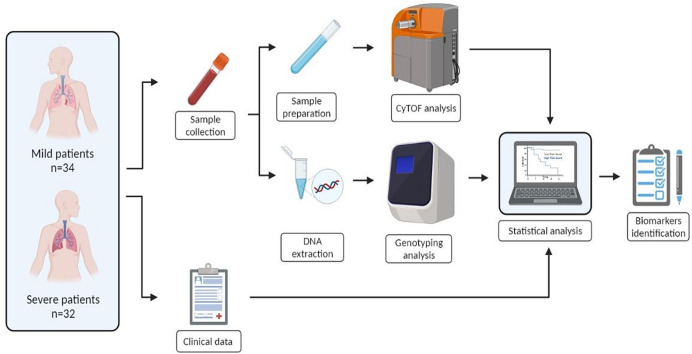

## 1 Introduction

Since its emergence in Wuhan in December 2019, the virus responsible for coronavirus disease 2019 (COVID-19), severe acute respiratory syndrome coronavirus 2 (SARS-CoV-2), has spread globally and become a world-threatening disease (1). According to the World Health Organization (WHO) report of 7 December 2022, the pandemic has exceeded 640 million cases and 6.6 million deaths worldwide (2). Since the onset of the pandemic, the search for biomarkers to correctly classify patients has been one of the major challenges for experts in this disease. Several biomarkers such as C-reactive protein (CRP), serum ferritin, D-dimer, and interleukin-6 (IL-6) have been studied for prognostic assessment of patients with COVID-19 pneumonia or simply for patient management (3). New biomarkers such as presepsin as a soluble CD14 subtype in sepsis patients have been continuously included (4).

The role of cytokines and chemokines has been associated with disease severity and clinical COVID-19 outcomes during all pandemic, suggesting that these molecules are the most promising biomarkers for patient management. Some inflammatory biomarkers have been reported to be significantly associated with an increased risk of developing severe COVID-19, such as procalcitonin (PCT), serum ferritin, CRP, IL-6, or erythrocyte sedimentation rate (ESR) (4). In addition, technologies combining flow and mass cytometry have improved multiple single-cell immune profiling in COVID-19 patients by revealing changes in both innate and adaptive immune cell subpopulations and their correlation with disease severity. There are reports of reduced frequencies in cell populations of severe COVID-19 patients in monocytes [particularly CD14^lo^ CD16^hi^ nonclassical monocytes (NC-Mo)], dendritic cells (DCs), and natural killer (NK) cells (5).

Due to the important role of the immune system and the immunological mechanisms that correlate with disease progression, we focus on the analysis using cytometry by time-of-flight mass spectrometry (CyTOF). This is a method that allows the simultaneous analysis of more than 40 cell markers without spectral overlap (5, 6).

There are controversial data regarding the best immunologic marker for COVID-19 monitoring. Recently, it has been reported that severe disease can be distinguished from moderate disease by systemic loss and dysfunction of M1-like pro-inflammatory monocytes, conventional DCs, and plasmacytoid dendritic cells (pDCs) (7). Moreover, in pediatric patients, it has been described that serum soluble CD25 and soluble CD163 levels have been described to be upregulated in the serum of SARS-CoV-2 patients (8), as well as increased levels of CD11c^+^. A CD16^hi^ population has been identified during the acute phase of infection and at disease severity in non-human primates (9).

There are also proteins such as the angiotensin-converting enzyme 2 (ACE2) whose high expression pattern is associated with an increased risk of severe infection and complications in COVID-19. In addition, a positive correlation of the upregulation of CD61 and CD163 with ACE2 expression has been found (10). The SARS-CoV-2 spike (S) protein binds to ACE2, which acts as an entry receptor. Once the receptor binds, the S protein is expressed and cleaved by the transmembrane protease serine 2 (encoded by TMPRSS2) (11). Therefore, these two genes have also come into focus as COVID-19 biomarkers, and the use of single-nucleotide polymorphisms (SNPs) as noninvasive biomarkers is readily available in routine clinical practice. For example, rs2285666 (G870A) has been suggested as the best option for the ACE2 polymorphism that modulates susceptibility to SARS-CoV-2 infection (12). Similarly, the TMPRSS2 gene has been reported to have several variants associated with susceptibility to COVID-19, mainly rs2070788, rs734056, rs12329760, rs2276205, and rs3787950 (13). Furthermore, rs2070788 is highlighted as a factor affecting COVID-19 severity. MX dynamin-like GTPase (MX1) has been highlighted as a critical responder in SARS-CoV-2 infection. Its expression is increased in COVID-19 patients and is strikingly associated with the increase in viral load (14). In addition, increased basal MX1 levels have been reported to correlate with SARS-CoV-2 infection, helping to identify the patient’s predisposition to severe disease (15).

Clinically, nearly 80% of COVID-19 cases are asymptomatic or have a mild form of the disease. However, approximately 13.8% and 6.1% are severe and critical, respectively, requiring hospitalization and even intensive care in the life-threatening cases (16). Our main objective is to demonstrate the role of various noninvasive biomarkers, especially cytometric and genetic biomarkers, that could predict or anticipate the most severe outcomes among these patients.

## 2 Materials and methods

### 2.1 Patients

A total of 66 (n = 34 mild and n = 32 severe) patients with a mean age of 52 years were recruited between 2020 and 2021. All clinical data [ferritin, D-dimer, CRP, troponin, lactate dehydrogenase (LDH)], symptoms (fever, anosmia, asthenia, dyspnea, long COVID, etc.), and intensive care unit (ICU) clinical follow-up (need for assisted ventilation, pneumonia, etc.) were included in the report; further details are described in **Table 1**. The following inclusion variables were taken into account for the severe group: (i) hypoxia with peripheral oxygen saturation (SpO2) ≤93% or partial pressure of oxygen/inspired oxygen fraction (PaO2/FiO2) >300 mmHg; (ii) respiratory rate (RR) ≥30 breaths/min; or (iii) ICU admission. The inclusion criteria for the mild group were (i) SpO2 >93%; (ii) the presence of nonspecific symptoms such as fever, fatigue, cough, or muscle pain, without hospitalization; or (iii) imperceptible symptoms during infection. In all patients, SARS-CoV-2 infection was confirmed by positive reverse transcription polymerase chain reaction (RT-PCR) or by positive IgM antibody test and at the same timeline after COVID-19 recovery. These inclusion criteria were revised periodically. For sample collection, only severe samples were collected in the ICU during the patient’s hospitalization, and the remaining groups were collected in primary assistance all collected post COVID-19 infection. Two tubes of peripheral blood were collected in ethylenediaminetetraacetic acid (EDTA) anticoagulant from each patient.

**Table 1:** Descriptive characteristic of the samples.

Variants	Patients
Age (years)	
<55	40 (60.6%)
≥55	26 (39.4%)
Sex	
Male	30 (45.5%)
Female	36 (55.5%)
Aggressiveness	
Mild	34 (51.5%)
Severe	32 (48.5%)
Flu Vaccine	
No	44 (66.7%)
Yes	21 (31.8%)
Missing	1 (1.5%)
Long COVID	
No	62 (93.9%)
Yes	1 (1.5%)
Missing	3 (4.5%)
Pneumonia	
No	33 (50%)
Yes	31 (47%)
Missing	2 (3%)
Systemic inflammatory response	
No	45 (68.2%)
Yes	18 (27.3%)
Missing	3(4.5%)

Long COVID, individuals with confirmed SARS CoV-2 infection with symptoms that last for at least 2 months and cannot be explained by an alternative diagnosis. Mo_Classical: Classical Monocytes.

Mo_Transitional: Transitional Monocytes.

Mo_Nonclassical Non classical Monocytes.

The study protocol was approved by the Research Ethics Committee of Granada (CEI-Granada) with internal code 1329-N-21. Written informed consent was obtained from all participants in accordance with the tenets of the Declaration of Helsinki.

### 2.2 Cytometry analysis

#### 2.2.1 Whole-blood sample processing

For flow cytometry analysis, blood samples were processed within 3 h after collection. Blood cells were fixed by withdrawing 700 μl of blood and adding 1 ml of Proteomic Stabilizer PROT1 (Smart Tube Inc., San Carlos, CA, USA) and incubating for 10 min at room temperature (RT). The blood was then frozen and stored at -80°C until staining.

After thawing at 4°C on a roller, samples were diluted in 13 ml of Thaw-Lysis Buffer 1X (Smart Tube Inc., San Carlos, CA, USA), filtered through a 100-µm pluriStrainer (pluriSelect Life Science, Leipzig, DE), and lysed in a roller for 10 min at RT. Cells were then pelleted, and leukocytes were washed with Maxpar^®^ Cell Staining Buffer (CST) (Fluidigm, San Francisco, CA, USA) and resuspended in 2 ml of CST. Blood samples were counted, aliquoting the same number of cells for each sample, 2.2 * 10^6^ cells/sample in this protocol. Then, all samples were pooled in a single tube and 1 µl of Fc block (BD Biosciences, Franklin Lakes, NJ, USA) was added and incubated for 10 min at RT. We then stained the surface antigens with the previously thawed surface antibody cocktail and incubated it at 4°C for 30 min. We then washed with CST and fixed with 1 ml of paraformaldehyde (PFA) 2% [stock 16% formaldehyde solution (ThermoScientific, Rockford, IL, USA)] and incubated for 10 min at RT. Then, we stained the DNA with iridium (Ir) solution and incubated overnight at 4°C with 1:2,000 Ir (125 μM) in Fix and Perm buffer. Samples were frozen and stored at -80°C until collection.

#### 2.2.3 Data acquisition

Cells stained for mass cytometry were thawed. Each sample was phenotyped at baseline using a 15-parameter monocyte and macrophage CyTOF panel (**Table 2**). For CyTOF acquisition, samples were washed in Maxpar^®^ Cell Acquisition Buffer (CAS). Prior to acquisition, 1 million cells/ml were resuspended in CAS containing EQ beads (1:10) and double filtered through 35-µm cell strainer cap tubes. Samples were acquired at a rate of 250–300 events per second on a Helios^®^ Mass Cytometer (Fluidigm, San Francisco, CA, USA).

**Table 2:** Antibody information.

Maxpar^®^ Human Monocyte/Macrophage Phenotyping Panel Kit			
Target	Clone	Source	Isotope
CD19	HIB19	Fluidigm	142Nd
CD11b	ICRF44	Fluidigm	144Nd
CD7	CD7-6B7	Fluidigm	147Sm
CD66	CD66a-B1.1	Fluidigm	149Sm
CD36	5-271	Fluidigm	152Sm
CD163	GHI/61	Fluidigm	154Sm
CD45	HI30	Fluidigm	156Gd
CD11c	Bu15	Fluidigm	159Tb
CD14	M5E2	Fluidigm	160Gd
CD16	3G8	Fluidigm	165Ho
CD38	HIT2	Fluidigm	167Er
CD206	15-2	Fluidigm	168Er
CD33	WM53	Fluidigm	169Tm
CD3	UCHT1	Fluidigm	170Er
HLA-DR	L243	Fluidigm	174Yb

#### 2.2.4 Data analysis

Raw data were normalized using MATLAB R2021a (MathWorks, Natick, MA, USA). We used GemStone™ version 2.0.45 software (Verity Software House, Topsham, ME, USA) and FlowJo version 10.8.1 (BD Biosciences, Franklin Lakes, NJ, USA) to analyze and clean CyTOF data. The normalized Flow Cytometry Standard File (FCS) files were transferred to GemStone™ software, which performs a standardized, automated, and unsupervised quality check (bead removal and selection of high-quality singletons). The software then analyzed different populations of leukocytes. Dimensionality reduction, clustering algorithm, and heatmap were performed using OMIQ data analysis software (OMIQ, Inc., Santa Clara, CA, USA). In addition, the Cen-se’ algorithm identifies related events based on measurement; we selected them and plotted them in a bivariate graph. Cen-se’ allows us to assess how accurately our model identifies these cells in our cell types. We can also examine cells that do not yet belong to any cell type and find out which measurements identify them (17).

### 2.3 Genetic analysis

Blood samples for genetic analysis were processed in the following 4–6 h after collection according to a protocol that depended on the subsequent analysis. For genotyping analysis, plasma was collected by centrifugation at 1,400g and 4°C for 10 min. After separation, plasma samples were frozen at -80°C until subsequent analysis.

The DNA extraction protocol was performed according to the manufacturer’s protocol of the Real Blood DNA Kit (Real life-science solutions, Valencia, Spain). All samples were standardized to 20 ng/μl using the Nanodrop 2000/2000c (ThermoFisher, Waltham, MA, USA) and had values between 1.8 and 2.0 A280/260. DNA genotyping was performed using the TaqMan^®^ Genotyping Master Mix (Applied Biosystems, Foster City, CA, USA), which contains all essential components (except probes, templates, and water) for polymerase chain reaction (PCR). Allelic discrimination assays were performed in a 7900HT Fast Real-Time PCR System (Applied Biosystems, Foster City, CA, USA). Results were analyzed using SDS software version 2.4 (Applied Biosystems, Foster City, CA, USA).

The selection of SNPs was performed according to *The National Center for Biotechnology Information* website in the most relevant data according to COVID-19 and genetic markers. In addition, only those SNPs with an allelic frequency greater than 20% in the minor allele (MAF) in the Caucasian population were selected from the Ensembl database (18). Finally, we selected *ACE2* (rs2285666), *MX1* (rs469390), and *TMPRSS2* (rs2070788) for the present analysis; see details of the probes in **Supplementary Table S1**.

### 2.4 Statistical analysis

Continuous flow cytometry variables were transformed into categorical variables using a binning strategy. Thus, flow cytometry variables were divided into two groups according to higher or lower expression based on the median value of the total number of patients. Categorical variables were then analyzed by chi-square test (χ^2^). Logistic regression analysis (either binary or multiple) was used to assess which of the genetic and/or flow cytometric factors might be determinant of COVID-19 risk. Odds ratios (ORs) and 95% confidence intervals (95% CIs) were calculated with p-value <0.05 as the criterion for significance. All analyses were performed with SPSS version 22 statistical package (IBM Corporation, Armonk, NY, USA) and GraphPad Prism version 8.2.1 (GraphPad Software, USA). The heatmap was created using the packages tidyr version 1.2.0, ggplot2 version 3.3.6, and forcats version 0.5.1 of R version 4.1.3. Before generating the heatmap, the samples were normalized using the min-max normalization method.

## 3 Results

### 3.1 Cytometry analysis

#### 3.1.1 General population description

**Figure 1:**
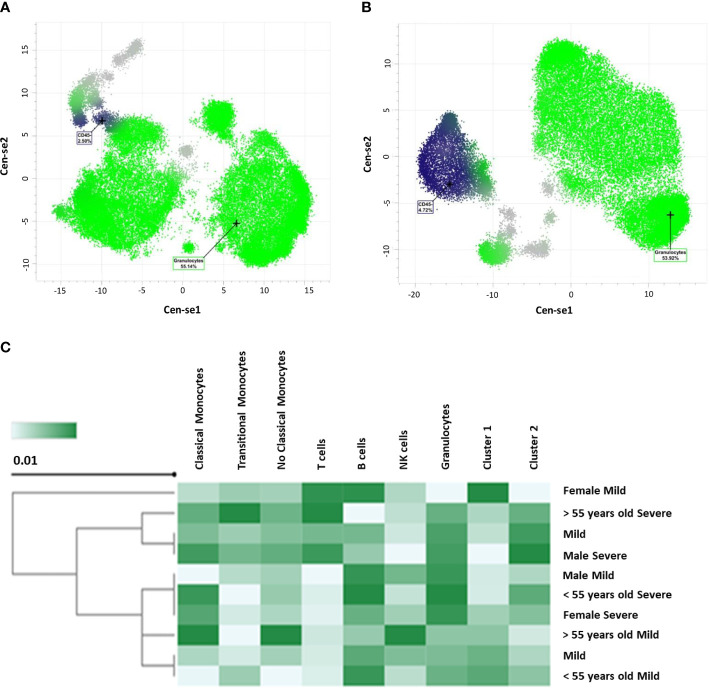
CD45^-^ represented by Cen-se’ algorithm with GemStone™ software. **(A)** Severe population of patients. **(B)** Mild population of samples. **(C)** Heatmap representation of both populations.

When performing a descriptive analysis of cytometry parameters in 66 patients (n = 34 mild and n = 32 severe) with a mean age of 52 years, we were able to summarize these markers as follows; see details in **Table 3**. As can be seen, CD45^-^ has a statistically significant value (p = 0.014) between mild and severe patients. Comparable results are reported for CD45^-^ when comparing Cen-se’ algorithm performed with GemStone™ software. Cen-se’ algorithm also showed that we can easily differentiate between severe (**Figure 1A**) and mild patients (**Figure 1B**) in the granulocyte population (**Figure 1**). A summary of the two populations is shown in a heatmap (**Figure 1C**).

**Table 3:** Analysis of cytometry variables in the main peripheral blood cell populations.

Variants	Mild	Severe	OR	95% CI	p-value
CD3^+^					
<22.51	16	17	0.784	0.298-2.063	0.622
≥22.51	18	15			
Granulocytes					
<54.41	18	15	1.275	0.485-3.354	0.622
≥54.41	16	17			
B Cells					
<2.78	15	17	0.697	0.264-1.837	0.464
≥2.78	19	15			
NK Cells					
<3.15	15	17	0.697	0.264-1.837	0.464
≥3.15	19	15			
CD45^-^					
<2.46	12	21	**0.286**	**0.104-0.787**	**0.014**
≥2.46	22	11			
Mo_Classical					
<4.82	17	16	1	0.381-2.626	1
≥4.82	17	16			
Mo_Transitional					
<0.07	14	17	0.618	0.233-1.636	0.331
≥0.07	20	15			
Mo_Nonclassical					
<0.18	15	17	0.697	0.264-1.837	0.464
≥0.18	19	15			
CD14^dim^					
<1.23	16	16	0.889	0.338-2.336	0.811
≥1.23	18	16			

In bold statistically significant values.

#### 3.1.2 Comparisons between severe and mild group of patients

**Figure 2:**
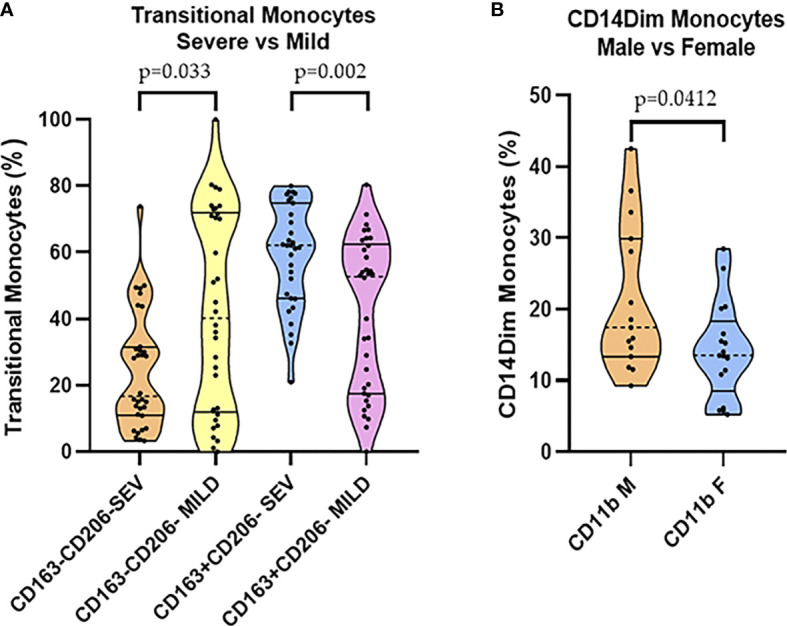
**(A)** CD163^+^/CD206^-^ and CD163^-^/CD206^-^ transitional monocyte comparisons in severe and mild groups. **(B)** CD11b CD14^dim^ monocytes in male vs. Female comparisons among the severe group.

CD163^+^/CD206^-^ frequencies were decreased in the mild group compared to that of the severe group in transitional monocytes (T-Mo), while CD163^-^/CD206^-^ frequencies were increased in the mild group compared to those in the severe group; see details in **Figure 2**. No differences were observed between the frequencies of T-Mo in CD163^+^/CD206^+^ and CD163^-^/CD206^+^ (**Supplementary Figure S1**). In addition, we found differences in the expression of CD11b in CD14^dim^-Mo in the severe group. These differences were decreased in the female (F in the figure) group (p = 0.0412) when clustering was performed in severe patients. Just to clarify, CD14^dim^-Mo is a monocyte population classified by GemStone™ software as an independent comparison with the classical monocyte (C-Mo) population (**Supplementary Figure S2**).

#### 3.1.3 Age comparisons

**Figure 3:**
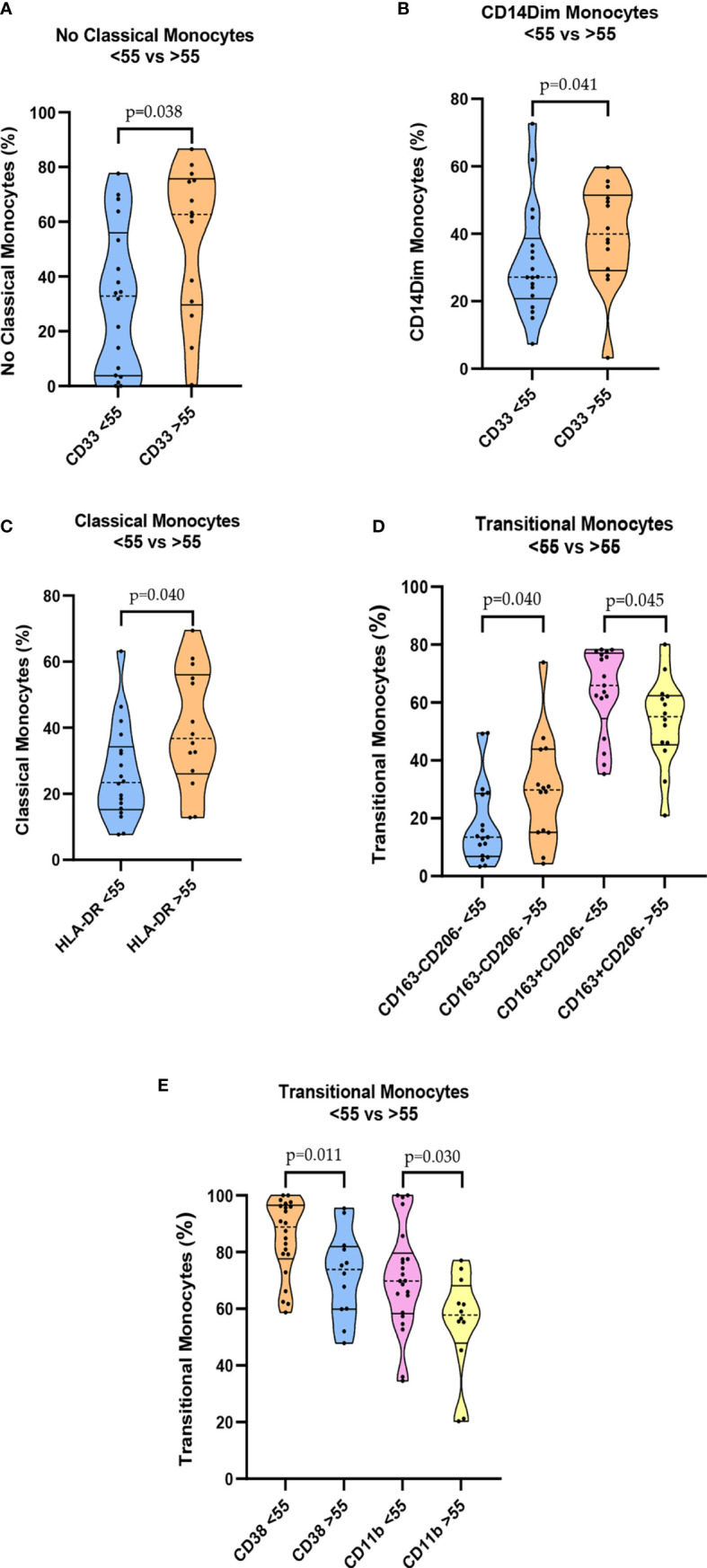
Cytometry analysis using age comparisons. Among the severe group: **(A)** Levels of CD33 NC-Mo cells. **(B)** Levels of CD33 CD14^dim^ monocytes. **(C)** Levels of HLA-DR classical monocytes. **(D)** Levels of CD163^+^/CD206^-^ T-Mo cells. Among the mild group: **(E)** Levels of CD38 T-Mo and CD11b T-Mo.

In the severe group comparisons, by age, patients ≥55 years old had an increase in some markers in the monocyte group compared with those in patients <55 years. For instance, the expression of CD33 was higher in NC-Mo, and this difference was also observed when total CD14^dim-Mo^ was analyzed in those older than 55 years (**Figures 3A, B** **)**. The expression of Human Leukocyte Antigen – DR isotype (HLA-DR) in C-Mo was also higher in the ≥55 group (**Figure 3C**). Surprisingly, T-Mo frequencies of CD163^+^/CD206^-^ were increased in the youngest, whereas CD163^-^/CD206^-^ were increased in the older (**Figure 3D**). When we compared the mild groups by age, we also found that T-Mo CD38 and CD11b were good biomarkers for stratifying patients, as shown in **Figure 3E**.

#### 3.1.4 Gender comparisons

**Figure 4:**
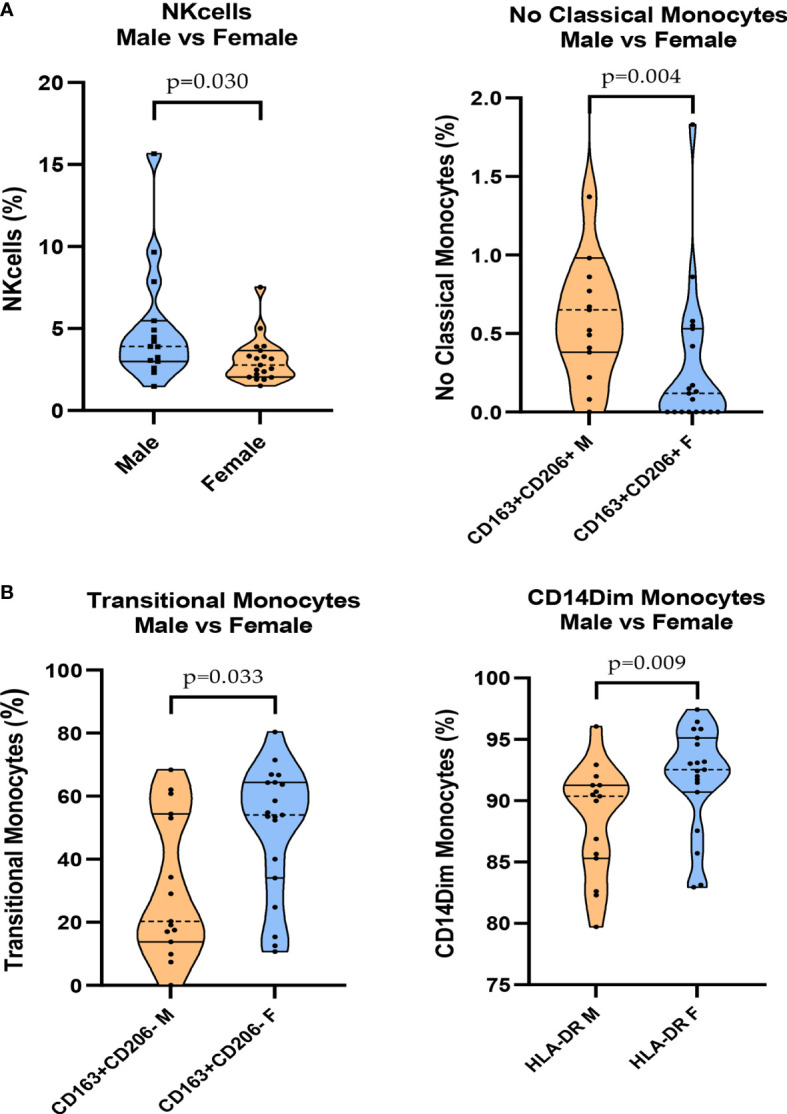
Mild group comparisons between men and women in cytometry analysis. **(A)** Levels of NK cells and CD163^+^/CD206^+^ NC-Mo cells. **(B)** Levels of CD163^+^/CD206- transitional monocytes and HLA-DR CD14^dim^ monocytes.

In the mild COVID group, we found a difference in NK cell expression. The frequencies were decreased in the female group compared to those in the male group (p = 0.030) (**Figure 4A**). The expression of CD11b was also higher in C-Mo (p = 0.043) (**Supplementary Figure S3**) and CD163^+^/CD206^+^ NC-Mo in the men (p = 0.004) (**Figure 4A**). The expression of HLA-DR in CD14^dim-Mo^ was lower (p = 0.009) and the frequencies in T-Mo of CD163^+^/CD206^-^ were decreased in the men (p = 0.033) (**Figure 4B**). A heatmap of these data is provided in **Supplementary Figure S4**.

**Figure 5:**
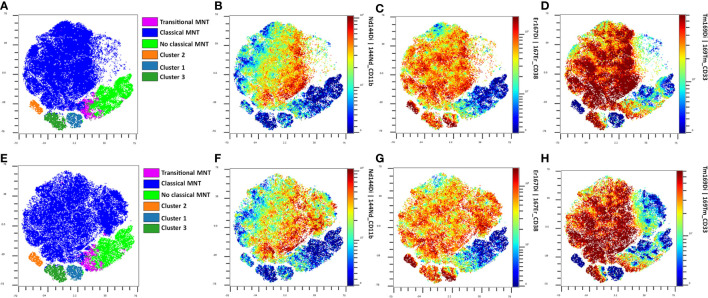
FlowSOM plots from the analysis of monocytes in mild patients. **(A)** FlowSOM plot showing the data from mild patients older than 55 years. **(B)** FlowSOM plot from mild patients under 55 years. **(C)** CD11b plot from mild patients older than 55 years. **(D)** CD11b plot from mild patients under 55 years. **(E)** CD38 plot from mild patients older than 55 years. **(F)** CD38 plot from mild patients under 55 years. **(G)** CD33 plot from mild patients older than 55 years. **(H)** CD33 plot from mild patients under 55 years.

In summary, when comparing mild vs. severe COVID-19 patients, we found that CD45^-^ (p = 0.014; OR = 0.286, 95% CI 0.104–0.787); T-Mo CD163^+^/CD206^-^ (p = 0.049; OR = 2.692, 95% CI 0.995–7.284), and CD14^dim^/CD33^+^ (p = 0.014; OR = 0.286, 95% CI 0.104–0.787) are the best options as biomarkers to discriminate between these populations. We confirm the role of CD38 and CD11b as good biomarkers for patient stratification by analysis with GemStone™ software; see **Figures 3E** and **5**. When compared with C-Mo, there is a clear difference. This difference becomes even greater when CD33 is included, although we found no significant changes in T-Mo CD33^+^ populations between severe and mild patients older or younger than 55 years. FlowSOM supports flow cytometry data in a self-organizing map (SOM) as an unsupervised clustering and dimensionality reduction technique, training a discretized representation of the input space. FlowSOM can be used either as a starting point for analysis or after manual gating for easy visualization of the results. Thus, it also provides information about subpopulations that may have been missed during the original manual gating (19).

### 3.2 Genetic analysis

We found that G carriers (*TMPRSS2* in rs2070788) have an increased risk (p = 0.02; OR = 3.37, 95% CI 1.18–9.60) of having severe COVID-19 compared to those with AA. In addition, it should be considered that other significances could not be observed due to the limited sample size for the present genetic analysis.

Interestingly, when combining genetic analysis with cytometry, we found three combinations with significant values, all in the *TMPRSS2* gene. Alone in the dominant model, it shows significant values comparing mild vs. severe patients (**Table 4**); now, this strength is reinforced when combining with CD45^-^, T-Mo CD163^+^/CD206^-^ and CD14^dim^/CD33^+^; details in **Table 5**.

**Table 4:** Genetic and COVID-19 aggressiveness comparisons.

Variants	Mild	Severe	p-value	OR	95% CI
*MX1*_rs469390_Dom					
AA	11	9	0.70	1.22	0.42-3.50
AG + GG	23	23			
*MX1*_rs469390_Rec					
GG	9	5	0.28	1.94	0.57-6.59
AG + AA	25	27			
*ACE2*_rs2285666_Dom					
CC	26	27	0.42	0.62	0.17-2.08
CT + TT	8	5			
*ACE2*_rs2285666_Rec					
CT + CC	31	30	0.69	1.45	0.22-9.30
TT	2	3			
*TMPRSS2*_rs2070788_Dom					
AG + GG	16	24	**0.02**	**3.37**	**1.18-9.60**
AA	18	8			
*TMPRSS2*_rs2070788_Rec					
AG + AA	30	25	0.27	0.47	0.12-1.81
GG	4	7			

Dom, dominant model; Rec, recessive model; OR, odds ratio; CI, confidence interval.

In bold statistically significant values.

**Table 5:** *TMPRSS2* and cytometry comparisons in COVID-19 aggressiveness.

Mild *vs*. Severe					
Variants	OR	95% CI	p-value	Omnibus test	R^2^ Nagelkerke
				(Step/Block/Model) *	
*TMPRSS2*_rs2070788_Dom	3.2	1.07-9.51	**0.036**	0.005	0.201
CD45^-^	0.3	0.10-0.85	**0.025**		
*TMPRSS2*_rs2070788_Dom	5.28	1.57-17.68	**0.007**	0.002	0.228
T-Mo CD163^+^/CD206^-^	4.34	1.34-13.96	**0.014**		
*TMPRSS2*_rs2070788_Dom	4.42	1.38-14.10	**0.012**	0.001	0.242
CD14^dim^/CD33^+^	0.22	0.07-0.679	**0.008**		

*All explanatory variables were added in one block and therefore have only one step. This means that the p-values are the same for the step, block, and model.

Dom, dominant model.

In bold statistically significant values.

## 4 Discussion

Although COVID-19 is a recent pathology, much of the research is focused on finding biomarkers for efficient diagnosis or patient stratification. There are data on the predictive ability of mortality in hospitalized patients using a score classification combining peripheral capillary oxygen saturation, albumin, D-dimer, and age (20). Others, focusing on neuroendocrine biomarkers such as copeptin, found an increase in severe cases (21). However, there are currently no conclusive data from clinical practice.

Here, we found the role of *TMPRSS2* (rs2070788) G allele carriers as an interesting and simple way to classify severe COVID-19 patients. This is not the first time that the role of this marker has been suggested, as it was previously mentioned by Akin et al. (22), who identified high levels of soluble *ACE2* as well as the *TMPRSS2* rs2070788 non-AA genotype and low aldosterone/renin ratio as independent factors for disease severity. *TMPRSS2* is the major host protease that enables cell entry of several coronaviruses and is highly expressed in lung and bronchial tissues. SARS-CoV-2 is known to utilize *ACE2* as a cleavage site for S-peak protein with the help of *TMPRSS2* (23). In contrast, in the present study, we found no differences between the severity of COVID-19 and the *MX1* or *ACE2* genes. *MX1* encodes a protein with antiviral activity against RNA and DNA viruses. Several studies have shown a high expression in COVID-19 patients, but we did not find a correlation (24). *ACE2* and especially several SNPs such as rs4646994 and rs2285666 have been suggested to correlate with COVID-19 susceptibility and/or disease severity, but controversial data on these two SNPs have been reported (25). Alimoradi et al. (26) and the present data suggest that there is no association between rs2285666 and COVID-19 severity.

In addition, the present analysis also exploited the power of CyTOF technology, which is currently one of the most powerful tools for immune phenotyping, allowing simultaneous and high-throughput quantification of more than 40 parameters at the single-cell level (5, 6). There are still few studies using this technology to characterize immune cell responses against SARS-CoV-2, as we do here (27). We focus on the role of monocytes, particularly the CD163^-^/CD206^-^ and CD163^+^/CD206^-^ populations, as the main markers for discriminating between severe and mild patients. A study conducted by Trombetta et al. (7) also suggests increased expression patterns of CD163 and CD206 as immunoregulatory markers in COVID-19. Elevated CD163 levels were positively correlated with *ACE2* expression, and CD163 indirectly contributes to the anti-inflammatory response. In addition, higher *ACE2* protein expression was found in severe COVID-19 disease, correlating with disease severity (10). CD163, due to its high expression in macrophages formed in response to tissue damage, is a potential inflammation biomarker and a therapeutic target (9). Here, we confirm its position in severe COVID-19 patients.

Here, we also reported the role of CD33 C-Mo associated with severe patients when compared with age. Previous data have shown that (CD33^-^ HLA-DMA^-^ CD14^+^) C-Mo and (CLEC10A^-^ S100A9^lo^) pDCs are involved in viral persistence and in the innate immune response against SARS-CoV-2 infection. Wang et al. (28) suggested that the enrichment of (CD33^-^ HLA-DMA^-^ CD14^+^) C-Mo may attenuate antigen presentation and antiviral immune response, which may also explain the present data in severe patients. Alberca et al. (29) also proposed a combination of the cell markers CD11b^+^ CD33^+^ HLA-DR^-^CD14^+^ and CD11b^+^ CD33^+^ HLA-DR^-^ CD66b^+^ as novel severity biomarkers for COVID-19. In the present study, we confirmed the presence of CD33^+^ CD11b^+^ cell markers in the blood of patients with severe disease. In addition, CD11b^+^ (macrophages and neutrophils) has also been described with high levels of cell infiltration in the lungs (9), which is common in severe patients. In addition, we found that CD14^dim^/CD33^+^ are good options as biomarkers for stratification of mild/severe COVID-19. Similarly, it has been previously reported that upregulation of C-Mo and, in particular, higher numbers of CD14^+^ CD33^+^ HLA-DR cells and S100A8/9/12 expressing C-Mo are present in severe/critical COVID-19 and sepsis (30). In the acute phase, Fahlberg et al. (9) showed a robust migration of CD16-expressing monocytes into the lung and described two subsets of interstitial macrophages (HLA-DR^+^CD206^-^) directly associated with plasma IL-6 levels. Furthermore, alveolar macrophages in acute lung injury with alveolar type II cell hyperplasia showed a characteristic phenotype (CD68, CD11c, CD14, CD205, CD206, CD123/IL3AR, and PD-L1) (31). As in our analysis in the severe population, we also found CD206 cell markers, in addition to those previously mentioned, in combination with CD80, which has been classified as an inflammatory monocyte subset not typically seen in healthy controls (32).

Moreover, CD45^-^ seems to be a good marker between mild and severe patients. Similar results were previously reported by Jin et al. (33) who suggested CD45 as a useful tool to discriminate between severe and non-severe cases. Furthermore, recent publications performed in healthy vs. COVID-19 patients reported that CD45 expression on leukocytes is altered in COVID-19 patients. This event is explained by the changes in signal transduction from binding to Toll-like receptor 4, changes in leukocyte subtypes, or maturation of cells during the infection process (34). It is reinforced by the important role that CD45 plays in autoimmune and oncological events, but also in viral infections (33).

According to gender, we found differences in NK and monocyte populations (C-Mo, T-Mo, and NC-Mo). In several studies, patient characteristics such as male sex, advanced age, and the presence of comorbidities have been associated with an increased risk of severe COVID-19 and ICU admission (35). However, this is the first time that differences in immunologic markers between men and women have been reported.

## 5 Conclusions

This analysis indicates the relevant role of several markers such as TMPRSS2, CD45^-^, CD163/CD206, and CD33 for COVID-19 aggressiveness. The optimal classification of severe or more aggressive COVID-19 patients could help clinicians to offer different stratifications and follow-up. This is relevant considering that the opposite has been described in other infectious diseases, where CD163 is decreased in C-Mo and T-Mo. Here, for the first time, we report a combination of markers that can be performed in blood and will help in this classification, although we must consider that one of the challenges of the present project is due to the limited size of the population. However, the use of high-throughput analysis such as CyTOF provides a lot of novel information in the present data.

## Data availability statement

The data presented in the study are deposited in the European Genome-Phenome Archive, accession number EGAD00010002445.

## Ethics statement

The studies involving human participants were reviewed and approved by 1329-N-21. The patients/participants provided their written informed consent to participate in this study.

## Author contributions

VA-R, SC-L, FM-B and PP-Q performed the experiments and data analysis. OS and JD-C performed cytometry analysis. LM-G and MA-C collected the related papers and drafted the manuscript. LM-G and MA-C participated in the design of the article. LM-G and MA-C wrote, and final proofed the manuscript. AA-M and MR-R performed the immunological revision and support of the article. SM-D, GL-T, SM-E and AC-V collected all the samples and updated clinical recording. LM-G and MA-C designed and supervised the study. SC-L and FM-B have statistical support. SM-D, CE-B, LM-G and MA-C obtained financial support. All authors contributed to the article and approved the submitted version.

## Glossary

**Table:**

ACE2	angiotensin-converting enzyme 2
CAS	Cell Acquisition Buffer
CEI	ethics committee
CI	confidence interval
C-Mo	classical monocytes
COVID-19	coronavirus disease 2019
CRP	C-reactive protein
CST	Cell Staining Buffer
EDTA	ethylenediaminetetraacetic acid
ESR	erythrocyte sedimentation rate
FiO2	inspired oxygen fraction
ICU	intensive care unit
IL-6	interleukin-6
LDH	lactate dehydrogenase
MAF	minor allele frequency
MX1	MX dynamin-like GTPase 1
NC-Mo	nonclassical monocytes
OR	odds ratio
PaO2/FiO2	partial pressure of oxygen/inspired oxygen fraction
PCR	polymerase chain reaction
PCT	procalcitonin
pDC	plasmacytoid dendritic cell
PFA	paraformaldehyde
RR	respiratory rate
RT	room temperature
RT-PCR	reverse transcription polymerase chain reaction
S	SARS-CoV-2 spike
SNP	single-nucleotide polymorphism
SpO2	peripheral oxygen saturation
T-Mo	transitional monocytes
TMPRSS2	transmembrane protease serine 2
WHO	World Health Organization
