# Supplementary material for: Relevance of TMPRSS2, CD163/CD206, and CD33 in clinical severity stratification of COVID-19
Source: Front Immunol. 2023 Mar 8;13:1094644. doi: 10.3389/fimmu.2022.1094644 (PMC10031647; doi:10.3389/fimmu.2022.1094644)
Supplement: Supplementary file 1 [file DataSheet_1.docx]

Supplementary Material


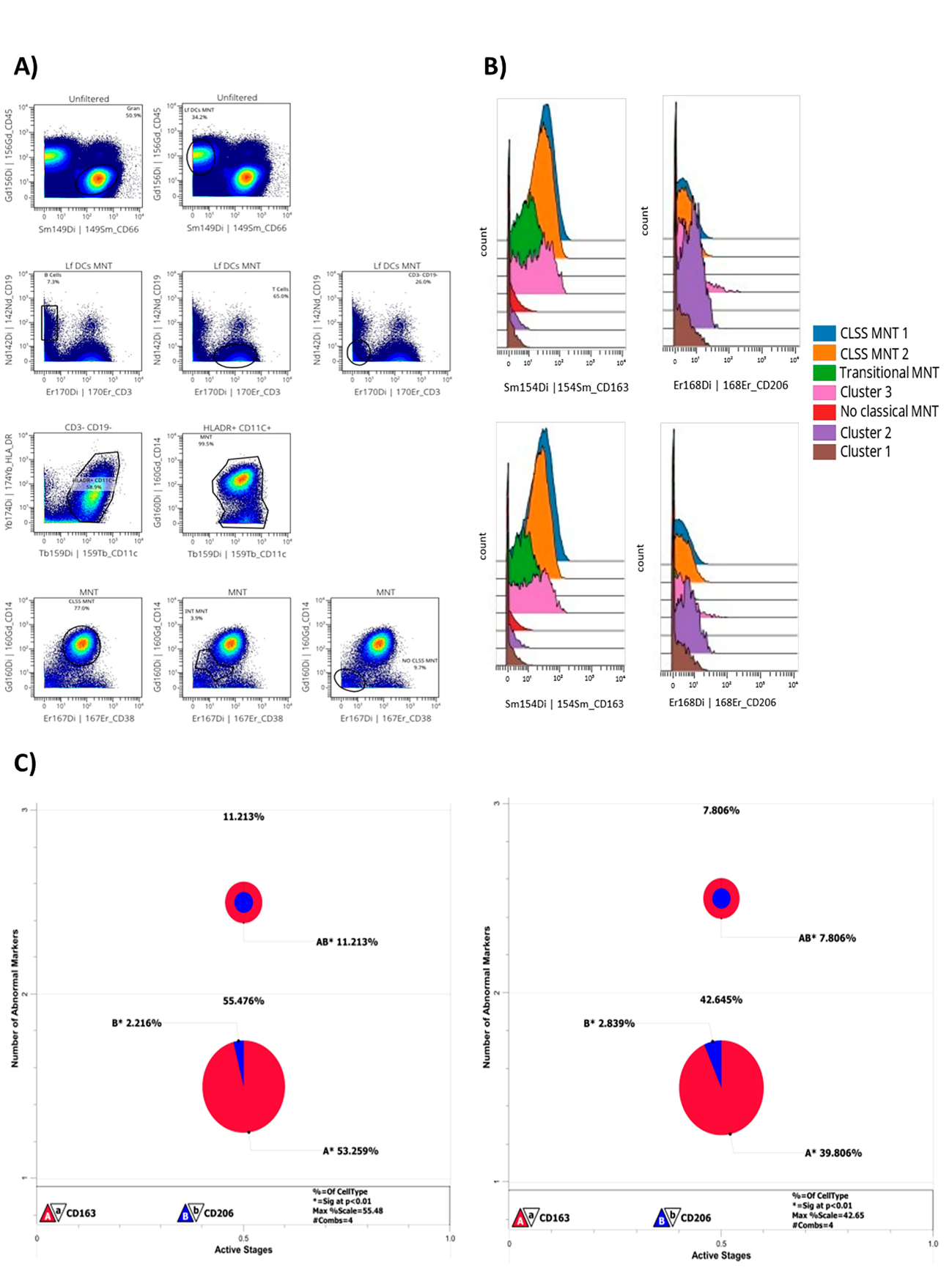


**Supplementary Figure 1.** Details of CD163/CD206 populations**. (A)**. Gating strategy for selecting all different populations of monocytes among severe patients. **(B).** Comparisons for CD163/CD206 between severe and mild patients in each cluster obtained by FlowSOM. **(C)**. Analysis by TriCOM in GemStone^TM^ of different markers expressions CD163 and CD206 in Mo_Transitional for severe patients (left) and mild (right). A abbreviate; on corresponds to CD163^+^/CD206^-^; B correspond to CD163^-^/CD206^+^; and AB correspond to CD163^+^/CD206^+^. Differences between percentages of these 3 populations is related to Mo_Transitional CD163^-^/CD206^-^.


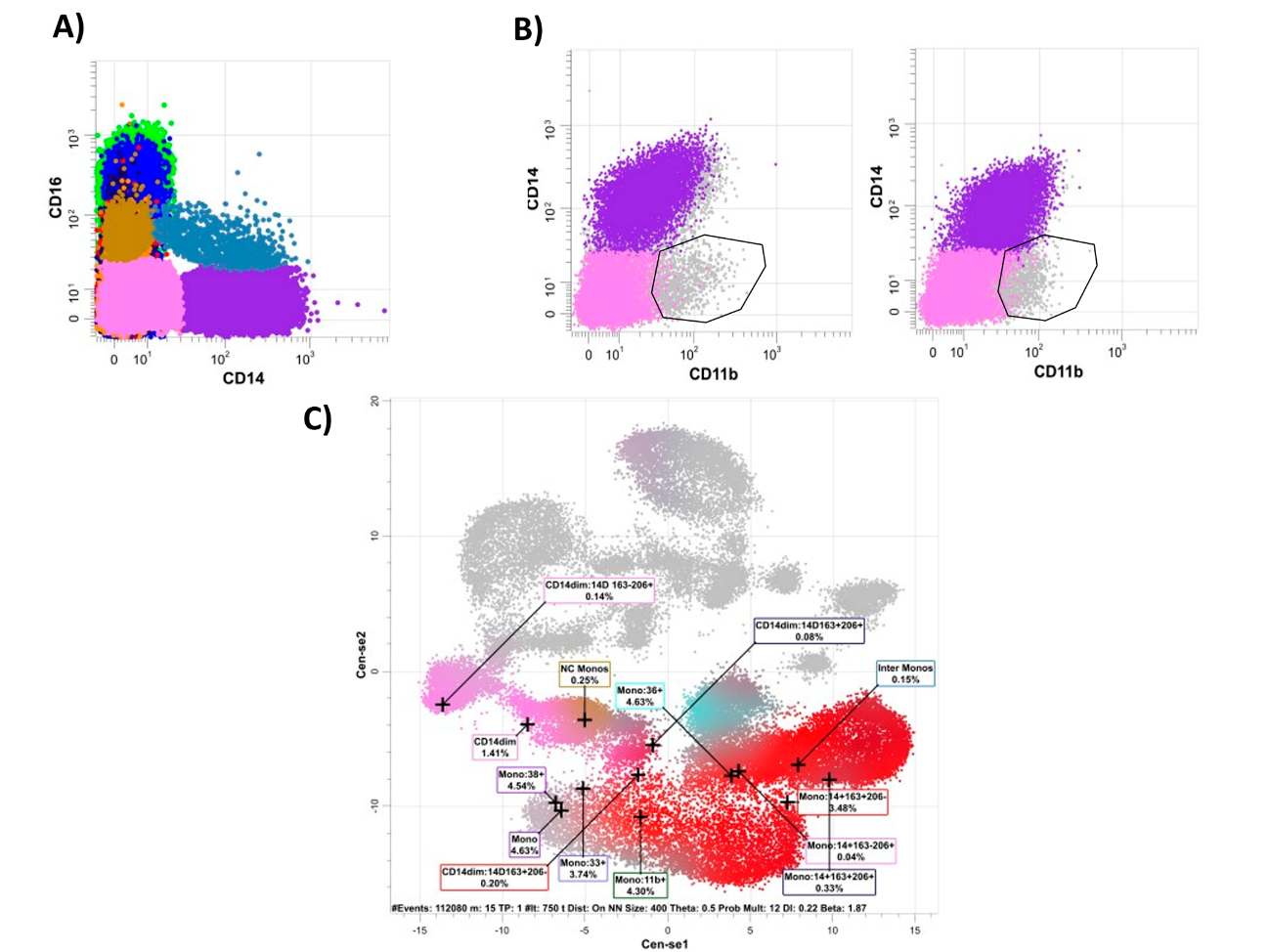


**Supplementary Figure 2.** Plots of monocyte subpopulations in severe patients **(A)**. In pink CD14dim monocytes (which is not totally negative for CD14 nor CD16). In blue Transitional Mo population. In brown Mo Non-Classical. Purple Mo classical. **(B)** Expression differences in CD11b between men and women in severe patients CD14^dim^ monocytes. **(C)**. Cen-se’ cluster of different monocytes in severe patients.


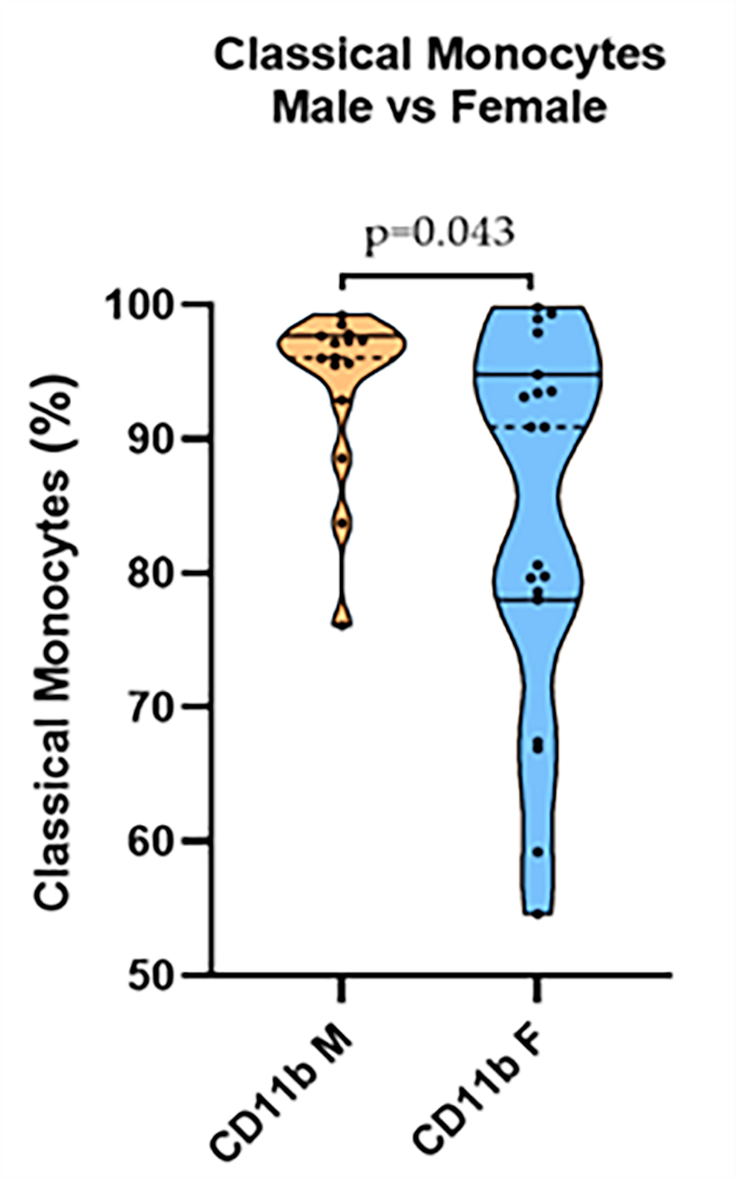


**Supplementary Figure 3**. CD11b levels comparisons in classical monocytes between males and females in the mild group.


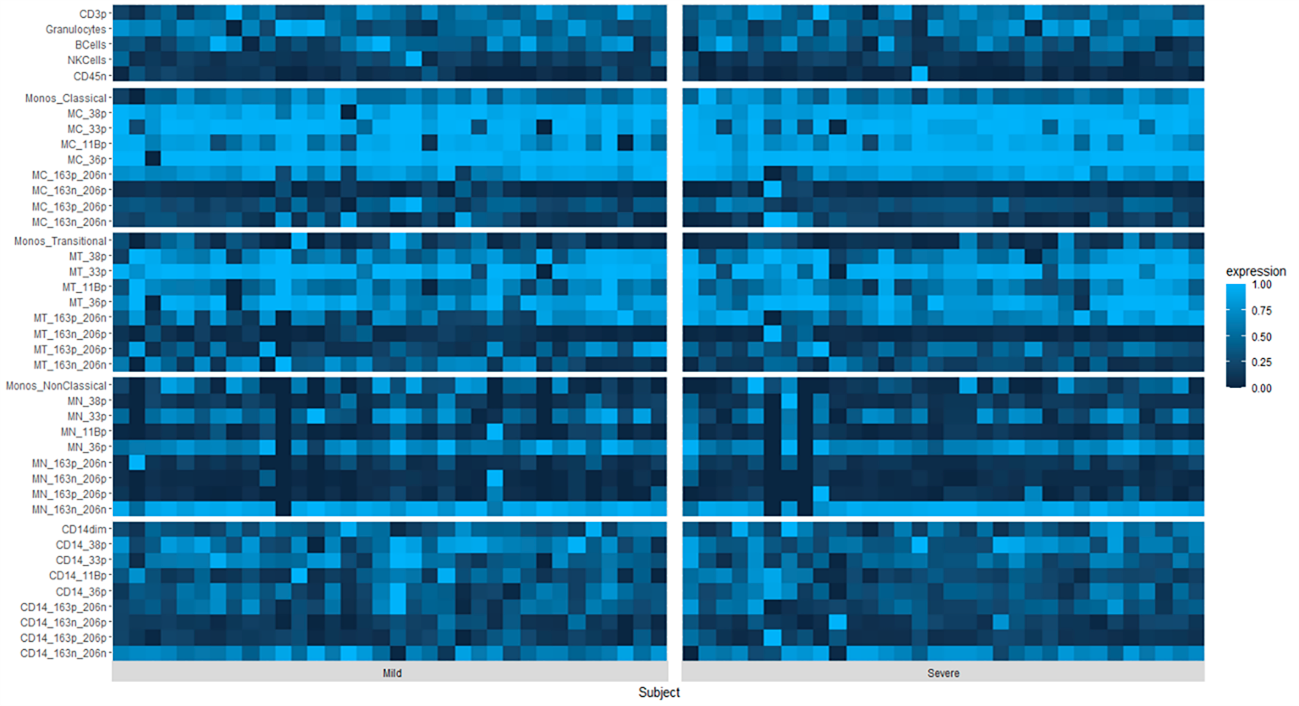


**Supplementary Figure 4.** Heatmap comparing mild versus severe group of patients. Abbreviations: n: negative; p: positive; MC: classical monocytes; MT: transitional monocytes; MN: Non classical monocytes.

**Supplementary Table 1:** Taqman^Ⓡ^ Probes details.

| **Gene ID** | **Probe ID** | **SNP ID** | **Alleles** | **EURO. MAF** | **SNP Type** | **Chromosome position** |
| --- | --- | --- | --- | --- | --- | --- |
| *ACE2* | C___2551626_1_ | rs2285666 | C/T | 0.23 | Transition Substitution,  Intron,  Intragenic | Chr.X: 15592225 on GRCh38 |
| *MX1* | C___2274997_10 | rs469390 | G/A | 0.41 | Transition Substitution,  Mis-sense Mutation,  Intragenic | Chr.21: 41446003 on GRCh38 |
| *TMPRSS2* | C___2592038_1_ | rs2070788 | G/A | 0.46 | Transition Substitution, Intron, Intragenic | Chr.21: 41470061 on GRCh38 |
